# Achieving equity for children and adolescents with perinatal HIV exposure: an urgent need for a paradigm shift

**DOI:** 10.1002/jia2.26171

**Published:** 2023-11-01

**Authors:** Jane Namangolwa Mutanga, Agnes Ronan, Kathleen M. Powis

**Affiliations:** ^1^ Department of Pediatrics and Child Health Livingstone Central Hospital Livingstone Zambia; ^2^ Paediatric Adolescent Treatment Africa Cape Town South Africa; ^3^ Department of Immunology and Infectious Diseases Harvard T.H. Chan School of Public Health Boston Massachusetts USA; ^4^ Departments of Internal Medicine and Pediatrics Massachusetts General Hospital Boston Massachusetts USA

**Keywords:** Adolescents, Children, HIV prevention, Testing, Vertical transmission

One and a half million children (0–14 years) were estimated to be living with HIV globally in 2022 by the Joint United Nations Programme on HIV/AIDS (UNAIDS), with 89% of these children residing in sub‐Saharan Africa [1]. It was also estimated that among children living with HIV, only 57% were accessing antiretroviral treatment (ART) compared to 77% of adults aged 15 years and older [2]. The inequity in access to ART between children and adults can be attributed to disproportionately poor uptake of HIV testing, gaps in timely ART initiation, low retention rates and poor adherence rates in the paediatric population compared to adults [3]. Fortunately, remarkable progress in the global scale‐up of ART for pregnant and breastfeeding persons living with HIV, along with improved risk‐based options for infant antiretroviral (ARV) prophylaxis, and increasing availability of pre‐exposure prophylaxis for persons at high risk of HIV acquisition, have led to marked declines in infant HIV acquisition [4]. UNAIDS reported a 58% reduction in the number of new HIV infections among children (0–14 years) between 2010 and 2022, offering hope of achieving sustainable development goal 3.3, targeting an end to AIDS as a public health threat by 2030 [4]. Concomitantly, recent data from the highest HIV burden settings demonstrate marked declines in vertical HIV transmission to as low as 3% in South Africa, 2.4% in Eswatini and 1.8% in Botswana[5].

However, over one million women and girls living with HIV around the world experience pregnancy annually, with 82% estimated to be accessing ART [6]. Therefore, although HIV acquisition in infancy has continued to decline, that decline has almost stalled in recent years, with 130,000 children estimated to have acquired HIV in 2022, compared to 140,000 in 2021 [7]. In addition, the number of children who are HIV‐exposed and uninfected (CHEU) has been steadily increasing by at least one million annually. UNAIDS estimates that the cumulative population of CHEU was approximately 16 million globally in 2022, and over 90% of these children reside in sub‐Saharan Africa [1, 8]. In South Africa, Eswatini, Botswana and Lesotho, where the prevalence of HIV is high and scale‐up of ART access in pregnancy is prioritised, over 20% of all infants born annually have had in utero exposure to HIV, and increasingly to maternal ART [9, 10].

Several studies have demonstrated health outcome disparities among CHEU compared to children without perinatal HIV exposure (children HIV‐unexposed and uninfected—CHUU), including high risk of mortality, morbidity, impaired growth and poorer neurodevelopment outcomes [11, 12, 13, 14, 15, 16, 17]. These studies demonstrate that in utero exposure to HIV can increase the risk of poor health outcomes across a child's life course, from early infancy into adulthood. Gaps remain in understanding the magnitude of these disparities and their underlying mechanisms, including the relative contributions from biological, social and/or structural processes. Furthermore, there is a lack of consensus on effective interventions to optimise the health and wellbeing of this growing population of children. Public health commitment and programming must go beyond HIV prevention and encompass a holistic approach that includes supporting children with perinatal HIV exposure at the highest risk of poor life course outcomes. Starting life HIV‐free is not a sufficiently high enough bar. CHEU at risk for poor outcomes require support to thrive to their fullest potential.

This supplement in the *Journal of the International AIDS Society* features studies and programmes that highlight gaps and describe interventions to improve health outcomes. In addition, opportunities to advocate for programming mitigating health outcome disparities are presented. Collectively, studies in this supplement motivate a more robust policy climate and response to optimizing outcomes of children and adolescents with perinatal HIV exposure with the intent of (1) identifying opportunities for basic science research to identify biological mechanistic pathways for observed health disparities; (2) informing the design of epidemiological studies and interventions investigating social and structural factors associated with increased risk of poor outcomes; (3) highlighting practical interventions to address observed disparities; (4) guiding health and educational policies and programming; and (5) ensuring that parents and caregivers of children affected by HIV have the knowledge needed to identify suboptimal outcomes and advocate for appropriate services.

Failure to diagnose HIV acquisition in a timely manner among infants and children increases the risk of mortality, infectious morbidity, poor growth and neurodevelopmental delays [18, 19, 20, 21, 22]. In the maturing HIV epidemic, the importance of client‐centred differentiated service delivery has been recognised as a necessary approach towards the goal of eliminating AIDS by 2030 [23]. Following timely diagnoses and initiation of ART, retention in care and achievement of viral suppression are paramount to optimizing the health of all persons living with HIV.

Retention in care has been particularly challenging among adolescents living with HIV [24, 25]. Internalised stigma has been noted as one of the factors associated with discontinuation of HIV care [26]. Harrison and colleagues report on a novel approach involving adolescents and young adults living with HIV in peer support groups comprised of similarly aged individuals living with other chronic illnesses [27]. In their clinic‐based pilot intervention called *The Better Together Program*, with peer leaders facilitating peer support group sessions, youth living with HIV reported value in learning about challenges encountered by their peers living with other chronic health conditions. Participation in the peer support groups for five or more sessions, compared to attending fewer sessions, was associated with higher self‐reported individual‐level resilience, a more positive attitude towards living with HIV, lower internalised stigma and a more positive self‐concept, measured using standardised instruments. This pilot provides a unique option for improving the mental wellbeing of adolescents living with HIV that may yield higher retention in care and sustained viral suppression.

de Beer and colleagues evaluated trends in hospitalisations due to infectious causes among children ≤3 years of age between 2008 and 2021. They evaluated perinatal HIV exposure as a surrogate measure of the HIV epidemic's impact on child health services in the Western Cape province of South Africa [28]. The authors analysed over 50,000 eligible hospitalisations and observed that children living with HIV accounted for a decreasing proportion of infectious‐cause hospitalisations, with CHEU comprising an increased proportion over time. Importantly, the Western Cape Provincial Health Data Centre's successful automated approach to linking unique maternal and child identifiers and data from various health encounters serves as a powerful model of how harmonised data systems can be leveraged to answer population‐level trends [29]. Such real‐world data can be useful in identifying implementation gaps and informing knowledge regarding the longer‐term health outcomes of CHEU.

Three studies in this supplement offer further insights on neurodevelopment outcomes for CHEU. Bulterys and colleagues report on findings from their Kenya‐based observational cohort of mother‐infant pairs where the Malawi Developmental Assessment Tool was administered to mothers who provided feedback on their 1‐year‐old infant's neurodevelopment in the domains of social, language, and fine and gross motor functioning [30]. The study evaluated maternal−infant pairs, half of whom were mothers living with HIV. Interestingly, while comparable social, fine and gross motor scores were found between infants who were HEU and those HIV‐unexposed uninfected (HUU), infants who were HEU had higher language scores. The authors also found that infants with in utero exposure to efavirenz had lower gross motor scores compared to infants with in utero exposure to dolutegravir, highlighting the importance of ensuring that registries are in place to monitor short‐ and long‐term health outcomes of in utero ARV exposure in order to identify the safest and most efficacious regimens for use in pregnancy.

A  trial in Eswatini conducted by Ruff and colleagues evaluated an intervention delivered by mentor mothers using the WHO's Nurturing Care Framework to improve nurturing care in high HIV burden settings [31]. Mother‐child pairs took part in the experiment in which the standard of care was compared to the intervention [31]. The authors found that a Nurturing Care Framework of activities delivered by mentor mothers can viably be integrated into antenatal care clinics and early intervention can reduce neurocognitive disparities. In addition, the authors observed that the mechanisms driving children's early language development occur through modifiable caregiving activities, including reading to infants and children. The provision of nurturing care in early life can positively influence optimal neurodevelopment in CHEU.

Powis and colleagues present educational achievement findings from children attending primary public school in the Botswana‐based FLOURISH study [32]. This prospective birth cohort study of children ages 8–12, of whom 75% were CHEU, demonstrated higher odds of lower academic performance, defined as a grade of “C” or less, among CHEU compared to those HUU in mathematics, science, English and overall. The association between being HEU and lower academic performance was attenuated after adjustment for maternal education, breastfeeding, low birth weight and child sex. Biological and socio‐demographic factors, including child sex and maternal education, appeared to contribute to this difference.

To better understand possible points of intervention to optimise neurodevelopmental outcomes of children with perinatal HIV exposure, the multifactorial elements associated with these outcomes must be considered. The commentary by Bulterys and colleagues offers insight into the possible biological and behavioural factors associated with neurodevelopmental outcomes among CHEU, from the in utero milieu to household and caregiver‐related factors [33]. The authors highlight that CHEU are at disproportionate risk of biological, social and household factors that may threaten their ability to achieve optimal maturation of their brain, immune system, and overall health and wellbeing. The authors advocate for structured early child development training for healthcare workers, equipping these gatekeepers with the ability to conduct rapid neurodevelopmental screening tests to identify children in need of specialised services to address neurodevelopmental delays while also promoting nurturing care among caregivers to mitigate the impact of poorer health outcomes.

The complex social milieu associated with sub‐optimal health outcomes among CHEU presents unique challenges for the design of optimal interventions. This is demonstrated in the South African‐based prospective cohort study by Le Roux and colleagues where pregnant persons and their children were followed through the child's first year of life, including people living with HIV and their CHEU from 2013 to 2017, with the aim of describing behavioural and socio‐economic factors associated with adverse child health outcomes [34]. The authors found that parental alcohol consumption, household intimate partner violence and household food insecurity were associated with poorer child growth and increased infectious morbidity. These risk factors were present at higher prevalence among CHEU compared to those HUU. To our knowledge, this is the first study to systematically evaluate evidence of HIV‐related syndemic interactions, at the maternal level or within the household, that potentiate adverse outcomes among CHEU compared to their unexposed peers.

The HIV field has been a champion in progressing global thought, action and capacity‐building towards models of healthcare that reflect the lived experiences, needs and preferences of affected individuals and communities [35]. However, infants and children cannot advocate for themselves. It is essential that parents be involved in setting the research agenda and service needs of children with perinatal HIV exposure. A commentary by Bukasa and colleagues discusses the importance of engaging mothers living with HIV in research plans and health communication strategies, and enabling mothers to inform the research agenda and contribute to health policies on behalf of their children [36]. It will be equally important to involve adolescents with perinatal HIV exposure in these conversations. However, adolescents who are HIV exposed and uninfected, when compared to adolescents living with HIV, are less likely to have received disclosure of their exposure status from their parents or caregivers. Davtyan and colleagues evaluated the role of internalised HIV stigma on willingness to disclose HIV status to CHEU. Data were derived from participants of the United States‐based Surveillance Monitoring for ART Toxicities (SMARTT) study, a study following outcomes of CHEU [37]. Among mothers living with HIV, disclosure was uncommon and mothers with higher scores for internalised stigma were less likely to disclose. This study demonstrates that internalised stigma must be mitigated among parents living with HIV to remove at least one barrier of HIV exposure disclosure to adolescents who are HIV exposed and uninfected.

Research funders, including the US National Institutes of Health (NIH), are prioritizing research to improve outcomes of children affected by HIV. Lee and colleagues, of the NIH, share current investments in the population of children affected by HIV, highlighting research gaps and outlining future areas of research focus [38]. The authors recommend that research priorities could be achieved more rapidly and sustainably by focusing on data science, data harmonisation and shared efforts between studies. Continued investment is required to expand research commitments that aim to identify biological, social and structural drivers of health outcome disparities. Lastly, given the large and growing population of CHEU, only a portion of whom are at increased risk of suboptimal outcomes, the authors stress that there exists an urgent need for the development of screening tools to identify high‐risk CHEU. The Viewpoint by Evans et al. discusses a comprehensive package of HIV‐specific and universal interventions that can be delivered throughout the first 1000 days of life [39]. These interventions can be delivered either to the mother or the infant and include early antenatal booking, infant ARV prophylaxis, nurturing care and healthy hygiene practices, among others.

In July 2022, the World Health Organisation, in partnership with UNAIDS and UNICEF, announced the Global Alliance to End AIDS in Children [3]. This strategic vision, designed to end AIDS in the paediatric population by 2030, has four pillars of focus, the first of which calls for optimised comprehensive, high‐quality treatment and care for infants, children and adolescents living with and exposed to HIV [3]. While much of this foundation has been laid for children and adolescents living with HIV, the work presented in this supplement highlights that merely celebrating an HIV‐free start to life for children and adolescents who are HEU is a short‐sighted objective. Collective and collaborative action must be taken to conduct impactful research, identify the multi‐faceted biological, social and structural factors that place children with perinatal HIV exposure at risk for poor outcomes, and transform findings into programming with continued monitoring and evaluation components.

## COMPETING INTERESTS

The authors declare that they have no competing interests.

## AUTHORS’ CONTRIBUTIONS

JNM, AR and KMP jointly drafted the editorial. All the authors read and approved the final version.

## DISCLAIMER

The views expressed in this article are the personal views of the authors and may not be understood or quoted as being made on behalf of or reflecting the position of the regulatory agency/agencies or organisations with which the authors are employed/affiliated.

## Data Availability

Data sharing not applicable to this article as no datasets were generated or analysed during the current study.
